# Anionic Polymerization of Styrene and 1,3-Butadiene in the Presence of Phosphazene Superbases

**DOI:** 10.3390/polym9100538

**Published:** 2017-10-21

**Authors:** Konstantinos Ntetsikas, Yahya Alzahrany, George Polymeropoulos, Panayiotis Bilalis, Yves Gnanou, Nikos Hadjichristidis

**Affiliations:** 1 Polymer Synthesis Laboratory, KAUST Catalysis Center, Physical Sciences and Engineering Division, King Abdullah University of Science and Technology (KAUST), Thuwal 23955, Saudi Arabia; konstantinos.ntetsikas@kaust.edu.sa (K.N.); yahya.alzahrany@kaust.edu.sa (Y.A.); georgios.polymeropoulos.1@kaust.edu.sa (G.P.); panagiotis.bilalis@kaust.edu.sa (P.B.); 2 Physical Sciences and Engineering Division, King Abdullah University of Science and Technology (KAUST), Thuwal 23955, Saudi Arabia; yves.gnanou@kaust.edu.sa

**Keywords:** anionic polymerization, phosphazene superbases, high vacuum technique, kinetic study, block copolymers

## Abstract

The anionic polymerization of styrene and 1,3-butadiene in the presence of phosphazene bases (*t*-BuP_4_, *t*-BuP_2_ and *t*-BuP_1_), in benzene at room temperature, was studied. When *t*-BuP_1_ was used, the polymerization proceeded in a controlled manner, whereas the obtained homopolymers exhibited the desired molecular weights and narrow polydispersity (Ð < 1.05). In the case of *t*-BuP_2_, homopolymers with higher than the theoretical molecular weights and relatively low polydispersity were obtained. On the other hand, in the presence of *t*-BuP_4_, the polymerization of styrene was uncontrolled due to the high reactivity of the formed carbanion. The kinetic studies from the polymerization of both monomers showed that the reaction rate follows the order of [*t*-BuP_4_]/[*sec*-BuLi] >>> [*t*-BuP_2_]/[*sec*-BuLi] >> [*t*-BuP_1_]/[*sec*-BuLi] > *sec*-BuLi. Furthermore, the addition of *t*-BuP_2_ and *t*-BuP_1_ prior the polymerization of 1,3-butadiene allowed the synthesis of polybutadiene with a high 1,2-microstructure (~45 wt %), due to the delocalization of the negative charge. Finally, the one pot synthesis of well-defined polyester-based copolymers [PS-*b*-PCL and PS-*b*-PLLA, PS: Polystyrene, PCL: Poly(ε-caprolactone) and PLLA: Poly(L-lactide)], with predictable molecular weights and a narrow molecular weight distribution (Ð < 1.2), was achieved by sequential copolymerization in the presence of *t*-BuP_2_ and *t*-BuP_1_.

## 1. Introduction

Anionic polymerization has been known for more than sixty years. An early report on anionic polymerization is traced back in the late 1920s by Ziegler and Bähr [1], but the first clear evidence for "living" anionic polymerization was reported by Michael Szwarc in the 1950s [2]. Since its discovery, it has emerged as the most powerful synthetic tool for the preparation of well-defined polymers with a narrow molecular weight distribution and controlled molecular characteristics (molecular weight, composition, microstructure and architecture) [3,4,5]. The polymerization proceeds via organometallic sites, carbanions (or oxanions) with metallic counterions. The most widely used initiators are organolithiums, soluble in organic solvents [6]. The ability of anionic polymerization to form well-defined macromolecules is mainly due, under appropriate conditions, to the absence of termination and chain transfer reactions [7,8].

Anionic ring opening polymerization (AROP) proceeds in the same manner but necessitates the use of catalysts in order to form nucleophilic species [9,10]. The most common initiators used for AROP are alkali metal derivatives such as hydrides, alkyls, aryls and mainly alkoxides of sodium, potassium and lithium [11]. Furthermore, the use of cryptands or crown ethers as catalysts has been reported [12], mainly for the AROP of heterocyclic monomers such as ethylene oxide, cyclosiloxanes, lactones etc. [13,14,15,16], but also for the anionic polymerization of styrene, butadiene, isoprene and methyl methacrylate [17,18,19,20]. These compounds form exceptionally stable cation inclusion complexes, in which the cation is completely surrounded by the ligand and hidden inside the molecular cavity. This leads to a high increase of the interionic distance in the ion pairs, resulting in higher polymerization rates, due to the suppression of the association between ion pairs in non-polar media [21]. Additionally, when the polymerization of dienes in non-polar solvent was performed in the presence of cryptands, high reaction rates were observed alongside with high vinyl content (up to 75%) [17,18].

**Scheme 1:**
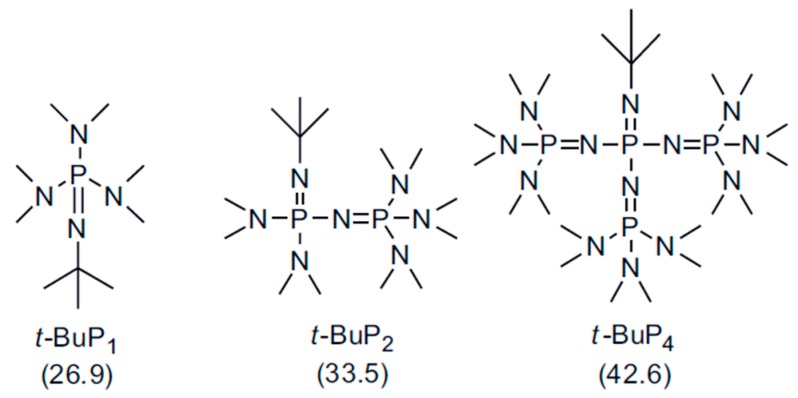
Chemical structures of the phosphazene bases *t*-BuP_1_, *t*-BuP_2_ and *t*-BuP_4_. The values in the parentheses indicate the *p*K_a_ in acetonitrile.

In addition to cryptates or crown–ether complexes, phosphazene superbases (PBs), a category of neutral Brönsted bases [22,23,24], have been extensively used as effective organic catalysts for the polymerization of several type of monomers, including epoxides [25,26,27,28], cyclosiloxanes [29,30], cyclic esters [31,32,33,34], cyclic carbonates [35]. The main feature of these non-nucleophilic bases is their high basicity (26 < p*K*_a_ < 43 in acetonitrile) (Scheme 1). There is an increase in basicity with an increased number of P atoms (P_1_ to P_4_), due to a rise of the delocalization of the charge on the conjugated phosphazenium cation [21]. Generally, phosphazene bases improve the nucleophilicity of the initiator/chain-end significantly by complexation with the counterion (e.g., proton or lithium cation), resulting in a rapid and controlled anionic polymerization [36]. Moreover, phosphazene bases are commercially available, chemically and thermally stable and soluble in non-polar and polar solvents [hexane, toluene and tetrahydrofuran (THF)] [22]. Phosphazene bases should be wisely chosen for each class of cyclic monomers in order to achieve the best compromise between polymerization rate and control. For instance, *t*-BuP_4_ is suitable for the polymerization of epoxides but leads to chain transfer reactions in the case of cyclic esters [37,38,39]. On the other hand the relatively mild ones (*t*-BuP_1_ and *t*-BuP_2_) provide better control in the polymerization of latter monomers [40].

To the best of our knowledge there are only two works reporting the use of organolithium compounds in combination with *t*-BuP_4_. Specifically, Möller et al. described the synthesis of poly(ethylene oxide) (PEO) utilizing *sec*-BuLi and *t*-BuP_4_ [41], while Förster et al. reported the in situ formation of *sec*-Bu^−^[(*t*-BuP_4_)Li^+^] complex in polar solvent, which served as the initiator for the synthesis of PB-*b*-PEO and PI-*b*-PEO [PB: polybutadiene and PI: polyisoprene] block copolymers [42]. Thus, in this work, we aim at expanding the scope of using phosphazene bases as organic catalysts through the investigation of the anionic polymerization of styrene and 1,3-butadiene in non-polar solvent via high vacuum techniques. Kinetic studies were conducted in the presence of *t*-BuP_1_, *t*-BuP_2_ and *t*-BuP_4_, using *sec*-BuLi as the initiator and benzene as the solvent. Furthermore, polyester-based copolymers [PS-*b*-PCL and PS-*b*-PLLA, PS: Polystyrene, PCL: Poly(*ε*-caprolactone) and PLLA: Poly(L-lactide)] were successfully synthesized through a one-pot methodology, combining anionic polymerization, phosphazene bases and ring-opening polymerization (ROP), leading to well-defined block copolymers with controlled molecular characteristics.

## 2. Materials and Methods 

### 2.1. Chemicals

1-*tert*-butyl-4,4,4-tris(dimethylamino)-2,2-bis[tris(dimethylamino)phosphoranyli-denamino]-2λ^5^,4λ^5^-catenadi-(phosphazene) (*t*-BuP_4_, 0.8 M in hexane, Sigma-Aldrich, Al-Khobar, Saudi Arabia), 1-*tert*-butyl-2,2,4,4,4-pentakis(dimethylamino)-2λ^5^,4λ^5^-catenadi-(phosphazene) (*t*-BuP_2_, 2.0 M in THF, Sigma-Aldrich) and *tert*-butylimino-tris(dimethylamino)phosphorane (*t*-BuP_1_, Sigma-Aldrich, 97%) were used as received. *sec*-Butyllithium (1.4 M in cyclohexane, Sigma-Aldrich) was diluted to the appropriate concentration in purified benzene, in a specific glass apparatus under high vacuum. Styrene (Sigma-Aldrich, 99%) was purified via consecutive distillations over CaH_2_ (Sigma-Aldrich, 95%) and dibutyl-magnesium (1 M in heptane, Sigma-Aldrich) and stored in pre-calibrated ampoules. 1,3-Butadiene (Sigma-Aldrich, 99%) was purified via consecutive distillations over *n*-BuLi, at −10 °C using ice/salt bath, prior addition to the polymerization reactor. Benzene and toluene (Sigma-Aldrich, 99.8%) were purified via distillation from CaH_2_ and stored in glass cylinders, under high vacuum. Ethylene oxide (Sigma-Aldrich, 99.5%) was used as the terminating agent and was purified by consecutive distillations over CaH_2_ and *n*-BuLi at 0 °C, with prior dilution to the appropriate concentration in purified benzene. *ε*-Caprolactone (Sigma-Aldrich, 97%) was dried over CaH_2_ twice and distilled under high vacuum. L-Lactide (Sigma-Aldrich, 98%) was recrystallized from ethyl acetate, dried under high vacuum overnight and finally dissolved in purified THF, prior the polymerization. Acetic acid (Sigma-Aldrich, 99.8%) (terminating agent) was stored under high vacuum and used as received.

### 2.2. Instrumentation

The number average molecular weight (*M_n_*) and the polydispersity index (Ð) were determined via size exclusion chromatography (SEC) equipped with an isocratic pump, Styragel HR2 and HR4 columns in series (300 mm × 8 mm), a refractive index detector and THF as the eluent, at a flow rate of 1 mL/min, at 30 °C. The calibration was performed using polystyrene standards (*M_p_*: 370 to 4,220,000 g/mol). Proton nuclear magnetic resonance (^1^H-NMR) spectroscopy measurements were carried out using CDCl_3_ (Sigma-Aldrich, 99.6%) on a Brücker AV-500 spectrometer. The obtained spectra were used to calculate the monomer conversion as well as the microstructure of the synthesized polybutadiene after integration of the corresponding chemical shifts.

### 2.3. Homopolymerization of Styrene and 1,3-Butadiene in the Presence of Phosphazene Bases (t-BuP_4_, t-BuP_2_, t-BuP_1_)

All polymerizations were anionic and carried out via high vacuum techniques, using custom-made glass reactors, equipped with break-seals for the addition of reagents and constrictions for the removal of aliquots [6]. A typical procedure is as follows (Table 1, Entry 2). 0.0215 mL (0.0302 mmol) of *sec*-BuLi was added to 50 mL of benzene. Then, 0.0151 mL (0.0302 mmol) of *t*-BuP_2_ was added and the solution was left under vigorous stirring for 1 h, at 25 °C. Afterwards, 0.82 mL of styrene was added and the reaction left to proceed at room temperature. At time intervals of 30 min, 1 h, 2 h, 4 h, 6 h and 8 h small aliquots were withdrawn from the solution in order to determine the conversion (^1^H-NMR) as well as the molecular weight and the polydispersity (SEC). Finally, the reaction was quenched by adding acetic acid and the solution precipitated in an excess of methanol. Conversion_(PS)_ = 100% after 2 h, theoretical number-average molecular weight of PS (*M_n_*, theoretical in Table 1, calculated from the feed ratio of monomer to initiator equal to 25,000 g/mol, number-average molecular weight of PS calculated by SEC *M_n,SEC_* = 72,000 g/mol, Ð = 1.19. The same synthetic protocol was followed using *t*-BuP_4_ and *t*-BuP_1_ for the polymerization of styrene, as well as for the polymerization of 1,3-butadiene

Sequential copolymerization of *ε*-caprolactone and L-lactide from the “living” PS using phosphazene bases (*t*-BuP_2_, *t*-BuP_1_).

A typical procedure is as follows. The “living” PS with a number-average molecular weight equal to 16,500 g/mol (Table 1, Entry 4) was synthesized in a similar way as described above, using 0.0302 mmol *sec*-BuLi, 0.0302 mmol of *t*-BuP_1_ and 0.5 mL of styrene. After 12 h of polymerization at 25 °C, the living carbanion was end-capped with 2–3 monomeric units of ethylene oxide, the benzene was removed under high vacuum and freshly distilled toluene was added. Then, 0.45 g of L-lactide was added and the polymerization left to proceed at room temperature. After 50 h the polymerization quenched by adding acetic acid and the mixture precipitated in an excess of methanol (Table 2, Entry 7). The white powder was collected and dried in a vacuum oven for two days. Conversion_(LLA)_ = 75% after 50 h, *M_n_,_PS_* = 16,500 g/mol, *M_n,PLLA_* = 11,500 g/mol. The same synthetic protocol was followed for the polymerization of *ε*-caprolactone.

## 3. Results and Discussion

The polymerization of styrene and 1,3-butadiene was performed in benzene, at a constant temperature of 25 °C, using one or 0.5 equivalent of the corresponding phosphazene superbases (*t*-BuP_4_, *t*-BuP_2_ and *t*-BuP_1_) with respect to the alkyllithium initiator (*sec*-BuLi). Two additional experiments were designed in the absence of phosphazene base, for comparison reasons. The molecular characteristics of the synthesized homopolymers, as well as the experimental conditions followed during the polymerization, are listed in Table 1.

**Figure 1:**
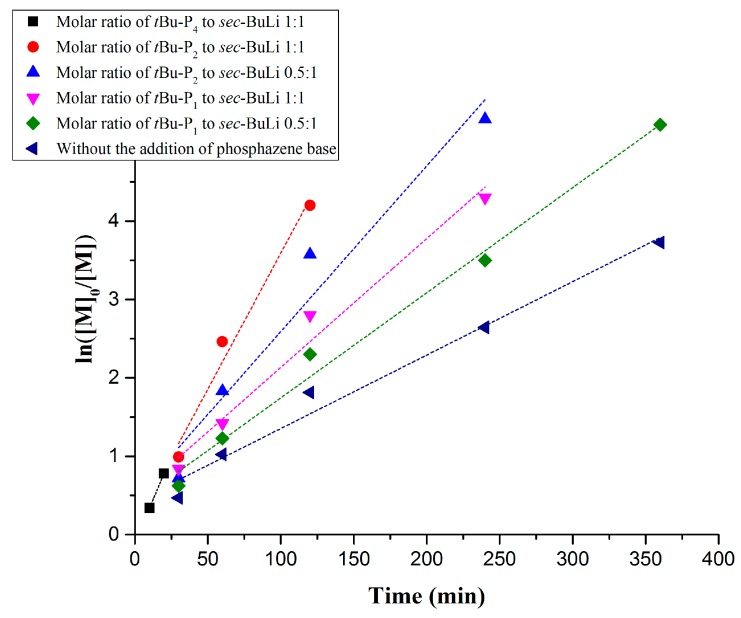
Kinetic plots of anionic polymerization of styrene in the presence of *t*-BuP_4_, *t*-BuP_2_, *t*-BuP_1_ and in the absence of phosphazene base.

One experiment (Table 1, Entry 1) was performed using [*t*-BuP_4_]/[*sec*-BuLi] in a molar ratio 1:1. In this case, high molecular weight (297,000 g/mol) with high polydispersity (Ð > 1.9) (Figure S1) was obtained in 20 min, suggesting that the basicity of *t*-BuP_4_ (*p*K_a_, _ACN_ = 42.6) leads to fast but uncontrollable polymerization of styrene (Figure 1, Black line). The big radius of the phosphazeniun lithium cation (~5 Å) [43,44] increases the interionic distance from the propagating anionic sites, generating extremely reactive carbanions, due to the delocalization of the positive charge over 17 positions in *t*-BuP_4_. We assume that during the polymerization either two active populations (aggregated non-active and active initiating species) coexist or the active species partially deactivated by the *t*-BuP_4_ and thus the produced polymers exhibit extremely high molecular weight and broad polydispersity. As a consequence, the viscosity of the solution was raised rapidly within 20 min, rendering the monitoring of the polymerization difficult. Further experiments are in progress to explain this complex phenomenon.

**Figure 2:**
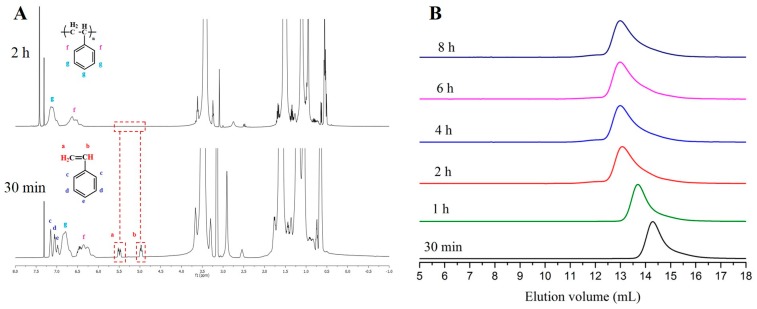
(**A**) ^1^H-NMR spectra after 30 min. and 2 h (100% conversion) {[*t*-BuP_2_]/[*sec*-BuLi]:1/1, styrene}; (**B**) SEC traces obtained from withdrawn aliquots at different time intervals during the [*t*-BuP_2_]/[*sec*-BuLi]:1/1 polymerization of styrene.

In order to gain better control over the polymerization rate of styrene in the presence of PBs, the weaker base *t*-BuP_2_, (*p*K_a_, _ACN_ = 33.5) was used. The kinetic study revealed that when *t*-BuP_2_ was added in a molar ratio 1:1, with respect to *sec*-BuLi (Table 1, Entry 2), the polymerization rate was significantly slower compared to *t*-BuP_4_ (same ratio between the base and the initiator) (Figure 1, Red line). Nevertheless, the conversion reached almost 100% after 2 h, as revealed by ^1^H-NMR analysis (Figure 2A). As it can be seen from Figure 2B, anionic polymerization of styrene in the presence of *t*-BuP_2_ was more controlled compared to *t*-BuP_4_, but still the homopolymer was characterized by relatively broad polydispersity (Ð > 1.2) and elevated molecular weights. In comparison with *t*-BuP_4_, the smaller radius of the phosphazeniun cation {[*t*-BuP_2_]Li^+^} (~3.3 Å) [43,44] and the delocalization of the positive charge over nine positions in *t*-BuP_2_, decreased the interionic distances between the cation and the propagating sites, resulting in a significant decrease of the propagating site reactivity. It is also probable that, as in the case of *t*-BuP_4_, part of the active centers, but to a lesser degree, are neutralized by the *t*-BuP_2_.

Decreasing the feed of *t*-BuP_2_ from one to 0.5 equivalent with regard to *sec*-BuLi (Table 1, comparison between entries two and three) a slightly slower polymerization rate was achieved (Figure 1, blue line). ^1^H-NMR analysis showed that 100% conversion was obtained after 4 h (Figure S2), while using [*t*-BuP_2_]/[*sec*-BuLi] in a molar ratio of 1:1 complete conversion was reached in only 2 h. This discrepancy is attributed to the differentiation in the equilibrium between *sec*-Bu^−^Li^+^ and *sec*-Bu^−^[(*t*-BuP_2_)Li^+^]. Even using a lower concentration of phosphazene base with respect to the alkyllithium initiator, the SEC traces (Figure S3) still exhibit high polydispersity (Ð > 1.19), but more controlled molecular weights.

**Figure 3:**
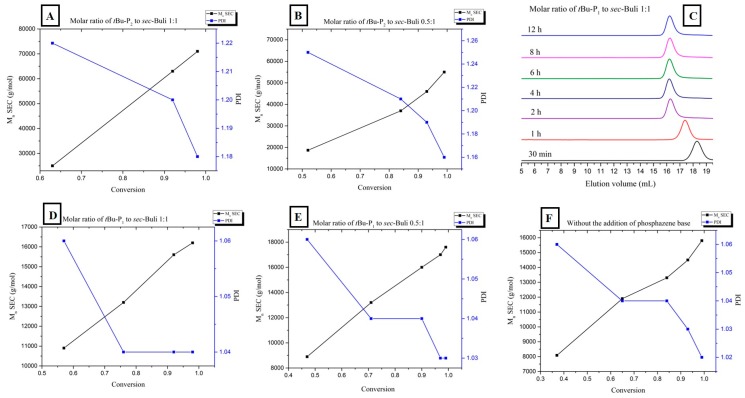
(**A**,**B**) Relationship between number-average molecular weight (black line) and polydispersity (blue line) vs. monomer conversion during the 1:1 and 0.5:1 [*t*-BuP_2_]/[*sec*-BuLi] polymerization of styrene; (**C**) SEC traces obtained from withdrawn aliquots at different time intervals during the [*t*-BuP_1_]/[*sec*-BuLi]: 1:1 polymerization of styrene; (**D**,**E**) Relationship between number-average molecular weight (black line) and polydispersity (blue line) vs. monomer conversion during the 1:1 and 0.5:1 [*t*-BuP_1_]/[*sec*-BuLi] polymerization of styrene; (**F**) Relationship between number-average molecular weight (black line) and polydispersity (blue line) vs. monomer conversion during the polymerization of styrene in the absence of phosphazene superbase.

For both experiments, the plot of the number of average molecular weight *M_n_* (measured by SEC) vs. conversion (measured by ^1^H-NMR) revealed a linear correlation, characteristic of the living character of the polymerization of styrene in the presence of PBs (Figure 3A,B). Moreover the polydispersity decreased considerably as the conversion reaches 100%.

To achieve the best compromise between the polymerization rate and the control over the molecular characteristics, two additional experiments were performed using the mild base *t*-BuP_1_ (*p*K_a_, _ACN_ = 26.9). For entries four and five (Table 1) the feed ratio of *t*-BuP_1_ was adjusted to one and 0.5, respectively, with regard to *sec*-BuLi. For both experiments the reaction rate appeared slower than the experiments where *t*-BuP_2_ was used (Figure 1, pink and green lines). Surprisingly, the SEC measurements from both homopolymers revealed excellent agreement between the calculated molecular weights and the targeted ones. In addition, as it can be seen in Figure 3C and Figure S4, the polymers exhibit very narrow molecular weight distribution (Ð < 1.04). These results are considered significant improvements over those obtained when *t*-BuP_4_ or *t*-BuP_2_ were used. When [*t*-BuP_1_]:[*sec*-BuLi] was adjusted to 1:1 and 0.5:1, ^1^H-NMR analysis revealed that in less than 4 h (Figure S5) and less than 6 h (Figure S6), 100% conversion was achieved, respectively. Based on the above findings, we can postulate that *t*-BuP_1_ suppresses the propagation rate, compared to *t*-BuP_2_ and *t*-BuP_4_, leading to homopolymers with controlled molecular characteristics.

From the kinetic study that was carried out (Figure 3D,E), it is evident that the number of average molecular weight increases linearly with conversion, confirming the complete absence of termination reactions. Additionally, the polydispersity remains below 1.05 over the entire conversion range.

In order to compare the results when *t*-BuP_4_, *t*-BuP_2_ and *t*-BuP_1_ were used; we investigated the kinetic study on the anionic polymerization of styrene using *sec*-BuLi without the addition of a superbase (Table 1, Entry 6). As expected, the reaction rate was significantly slower when no catalyst was present (Figure 1, dark blue line). The anionic polymerization of styrene produced a homopolymer with extremely narrow polydispersity (Ð < 1.02) exhibiting the desired molecular weight (Figure S7). Complete monomer conversion was observed after 6 h (Figure S8), while the linear dependence of the *M_n_* with regard to the monomer conversion (Figure 3F) indicated the livingness of the method.

It is well known that the typical microstructure of polybutadiene prepared via anionic polymerization in hydrocarbon solvents is 92 wt % 1,4- and 8 wt % 1,2-microstructure [45,46,47]. On the other hand, the addition of polar additives such as ethers or amines, prior to the polymerization of butadiene, results in polybutadiene with a high 1,2-microstructure [39]. To the best of our knowledge, only Förster et al. reported the addition of a superbase (*t*-BuP_4_) prior to the polymerization of a diene [42]. Since authors used tetrahydrofuran as a solvent to synthesize PB-*b*-PEO and PI-*b*-PEO block copolymers, the effect of *t*-BuP_4_ on the vinyl content could not be investigated. The determined microstructure (89 wt % 1,2- and 11 wt % 1,4-microstructure) was identical to the values reported in the literature regarding the polymerization of butadiene in polar solvents without phosphazene bases [8].

**Figure 4:**
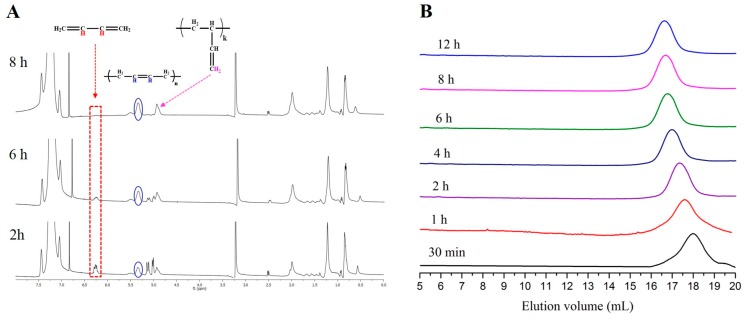
(**A**) ^1^H-NMR spectrum after 2 h, 6 h and 8 h (100% conversion) {[*t*-BuP_2_]/[*sec*-BuLi]:1/1, 1,3-butadiene}, indicating the different polybutadiene microstructures; (**B**) SEC traces obtained from withdrawn aliquots at different time intervals during the [*t*-BuP_2_]/[*sec*-BuLi]:1/1 polymerization of butadiene.

Based on that context, we were inspired to investigate the influence of the PBs on the microstructure of polybutadiene when the polymerization is performed in a hydrocarbon solvent. As revealed by ^1^H-NMR analysis, when *t*-BuP_2_ or *t*-BuP_1_ were added prior the polymerization, the 1,2-microstructure was increased as high as 45% and 43% (Figure 4 and Figure S9), due to the delocalization of the negative charge caused by the formation of PB^−^[(*t*-BuP_2_)Li^+^] or PB^−^[(*t*-BuP_1_)Li^+^] complex, respectively. On the contrary, when the polymerization occurred in the absence of a superbase, the synthesized polybutadiene was characterized by 90 wt % 1,4- and 10 wt % 1,2-content (Figure S10). The above-mentioned deviation in the content of the 1,2-microstructure of the synthesized polybutadiene indicates that superbases can also affect the microstructure as common polar additives [48,49,50].

**Figure 5:**
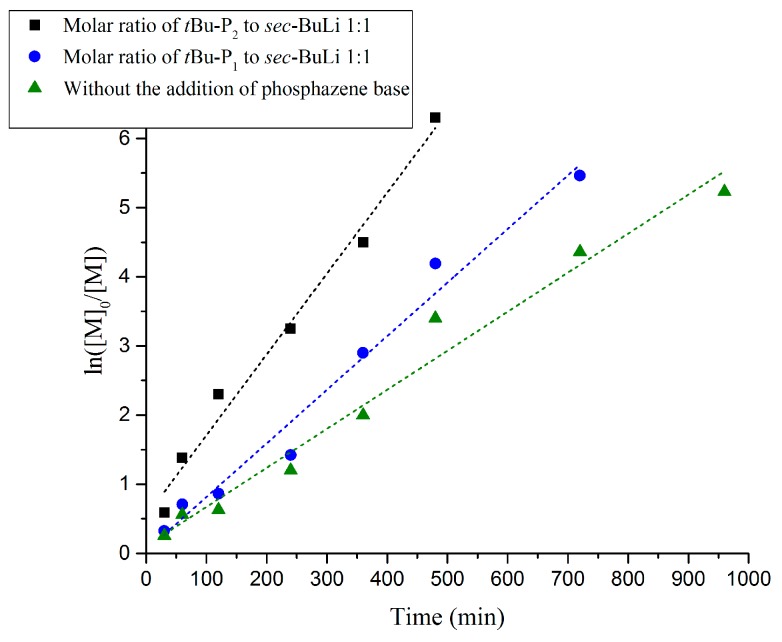
Kinetic plots of anionic polymerization of 1,3-butadiene in the presence of *t*-BuP_2_, *t*-BuP_1_ and in the absence of phosphazene base.

**Figure 6:**
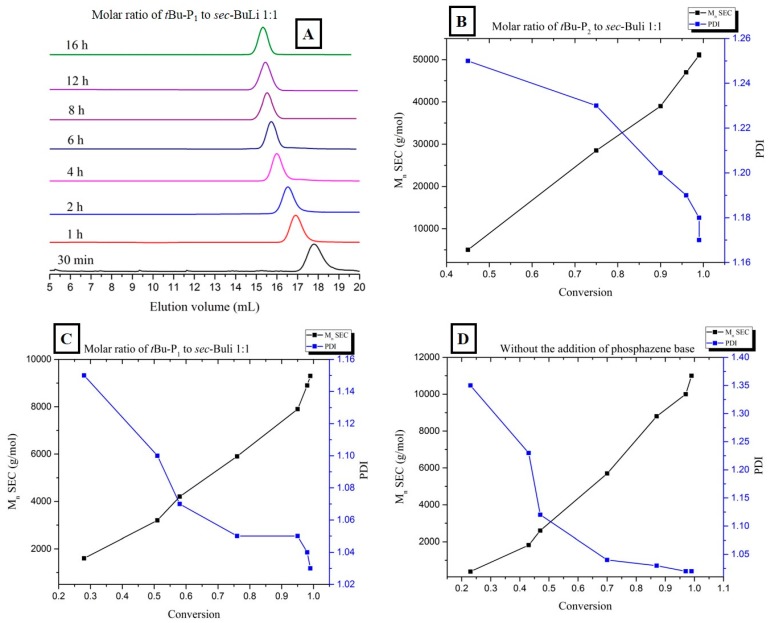
(**A**) SEC traces obtained from withdrawn aliquots at different time intervals during the [*t*-BuP_1_]/[*sec*-BuLi]:1/1 polymerization of butadiene; (**B**,**C**) Relationship between number-average molecular weight (black line) and polydispersity (blue line) vs monomer conversion during the 1:1 [*t*-BuP_2_]/[*sec*-BuLi] and [*t*-BuP_1_]/[*sec*-BuLi], polymerization of butadiene, respectively; (**D**) Relationship between number-average molecular weight (black line) and polydispersity (blue line) vs. monomer conversion during the polymerization of 1,3-butadiene in the absence of phosphazene superbase.

The reaction rate of the polymerization of butadiene, as confirmed by kinetic studies, also follows the order of [*t*-BuP_2_]/[*sec*-BuLi]:1/1 >> [*t*-BuP_1_]/[*sec*-BuLi]:1/1 > *sec*-BuLi (Figure 5). As discussed above, the higher basicity of *t*-BuP_2_ results in faster but uncontrolled polymerization (Table 1, Entry 7), whereas the homopolymer is characterized by high polydispersity (Ð > 1.17) and elevated molecular weights (Figure 4B). When the mild *t*-BuP_1_ was used, (Table 1, Entry 8), the homopolymer was characterized by excellent control over the molecular weight and very narrow polydispersity (Ð < 1.03) (Figure 6A). Finally, when the polymerization was performed without the addition of a superbase, the SEC traces of the synthesized polybutadiene (Figure S11) showed a narrow molecular weight distribution (Ð < 1.02) and excellent control over the molecular weight.

Adjusting the feed ratio of [*t*-BuP_2_]/[*sec*-BuLi] to 1/1 (Table 1, Entry 7), ^1^H-NMR analysis revealed complete monomer conversion after 8 h (Figure 4A). On the contrary, when *t*-BuP_1_ was added prior to the polymerization (Table 1, Entry 8) or *sec*-BuLi was used without the addition of a superbase (Table 1, Entry 9), complete conversion was reached after 12 and 16 h, respectively, as a result of the slower polymerization rate (Figures S9 and S10).

Finally, as can be seen in Figure 6B–D, a linear dependence of the apparent molecular weight *vs* the monomer conversion was obtained for all experiments, while a narrow molecular weight distribution is observed throughout the conversion spectrum.

To conclude, the addition of the superbases prior the polymerization of styrene and/or 1,3-butadiene speeds up the reaction rate due to the generation of highly reactive anionic species through complexation with the lithium cation. On the other hand, our kinetic study showed that using the low basicity *t*-BuP_1_, the polymerization rate seems still enhanced as compared to the conventional metal-based initiating systems, whereas well-defined homopolymers were prepared.

The one-pot synthesis of PS-*b*-PLLA and PS-*b*-PCL using the “living” PS, synthesized in the presence of *t*-BuP_1_ and *t*-BuP_2_, was also examined. The molecular characteristics of the obtained copolymers, as well as the experimental conditions followed during the polymerizations, are listed in Table 2.

It is well-known that phosphazene bases (BEMP, *t*-BuP_1_ and *t*-BuP_2_) catalyze the ring-opening polymerization of *ε*-CL and LLA using either various alcoholic initiators or hydroxyl end-functionalized polymers in different solvents (toluene, THF) [31,32,33].

**Scheme 2:**
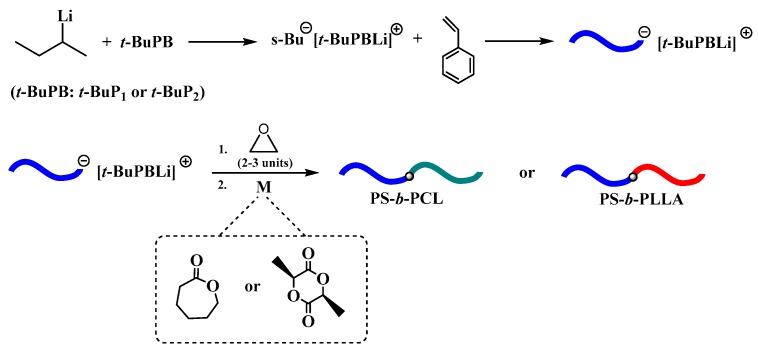
Anionic synthesis of polystyrene in the presence of phosphazene bases and the subsequently ring-opening polymerization of L-lactide and ε-CL.

Initially, the ROP of ε-CL and LLA was examined using the macroinitiator PS^−^[(*t*-BuP_1_)Li^+^] or PS^−^[(*t*-BuP_2_)Li^+^] (Table 2, Entries 1–4), but as it was expected in all cases no polymerization obtained. This is attributed, probably, to the deactivation of the highly basic and nucleophile carbanion from the acidic hydrogen of CL or LLA. Thus, the transformation of the carbanion to the less nucleophilic alkoxide is necessary [51]. For this reason, after the formation of PS^−^[(*t*-BuP_1_)Li^+^] or PS^−^[(*t*-BuP_2_)Li^+^], the living carbanion was end-capped with 2-3 monomeric units of ethylene oxide, to generate PSO^−^[(*t*-BuP_1_)Li^+^] or PSO^−^[(*t*-BuP_2_)Li^+^] respectively (Scheme 2). Afterwards, the benzene was removed and freshly distilled toluene was added prior to the second monomer addition [33]. Following this strategy, it was feasible to obtain well-defined block copolymers of PS-*b*-PLLA and PS-*b*-PCL.

**Figure 7:**
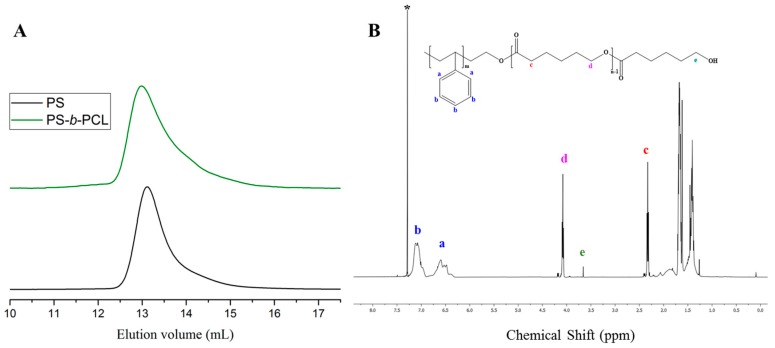
(**A**) SEC traces of PS (black line) synthesized by [*t*-BuP_2_]/[*sec*-BuLi]:1/1 and of the corresponding PS-b-PCL (green line); (**B**) ^1^H-NMR spectrum of the final diblock copolymer in CDCl_3_.

In the case of the ROP of L-lactide in toluene at room temperature, using PSO^−^[(*t*-BuP_2_)Li^+^] as a macroinitiator (Table 2, Entry 5), SEC analysis confirmed clean chain-extended block copolymer (PS-*b*-PLLA) free from unreacted macroinitiator contamination and showed excellent control of molecular weight (Figure S12A). The polymerization of L-lactide was very fast, thus complete conversion (100%) was reached after 20 min. The ^1^H-NMR spectrum (Figure S12B) reveals the characteristic signals of both blocks, while through end-group analysis, the degree of polymerization of L-lactide was calculated equal to DP_PLLA_ = 110. Additionally, the same macroinitiator {PSO^−^[(*t*-BuP_2_)Li^+^]} was also used for the ROP of *ε*-CL (Table 2, Entry 6) in the same conditions. As indicated from the ^1^H-NMR analysis, the polymerization rate of *ε*-CL was slower compared to the polymerization of L-lactide, since conversion of ~70% was achieved at 17 h. Figure 7B shows the ^1^H-NMR spectrum of the final PS-*b*-PCL, with DP_PCL_ = 92.

**Figure 8:**
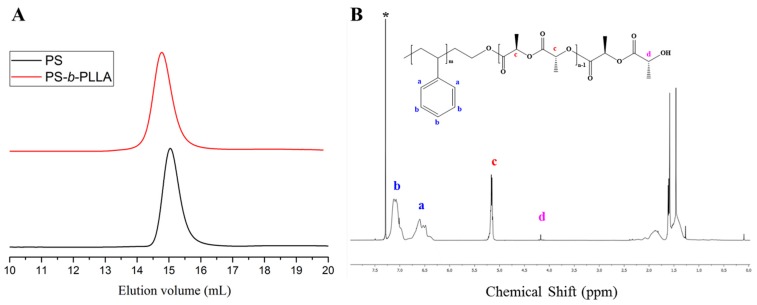
(**A**) SEC traces of PS (black line) synthesized with [*t*-BuP_1_]/[*sec*-BuLi]:1/1 and the corresponding PS-b-PLLA (red line); (**B**) ^1^H-NMR spectrum of the final diblock copolymer in CDCl_3_.

Furthermore, the polymerization of L-lactide and *ε*-CL using PSO^−^[(*t*-BuP_1_)Li^+^] as a macroinitiator was examined in toluene, at room temperature. It has been reported [32] that using the weaker phosphazene base, *t*-BuP_1_, as the catalyst and 1-pyrenebutanol as the initiator, PLLA can be obtained with controllable molecular weight and low polydispersity index (Ð ≈ 1.06). Similar results were observed in the case of ROP of L-lactide utilizing PSO^−^[(*t*-BuP_1_)Li^+^] as the macroinitiator (Table 2, Entry 7). The SEC and ^1^H-NMR analysis (Figure 8A,B) verified the successful synthesis of PS-*b*-PLLA with low polydispersity (Ð ≈ 1.07) and DP_PLLA_ = 84, through end-group analysis. The conversion was calculated to be ~75% after 50 h and the polymerization rate was much slower than in the case of PSO^−^[(*t*-BuP_2_)Li^+^].

Finally, the ROP of *ε*-CL utilizing PSO^−^[(*t*-BuP_1_)Li^+^] as the macroinitiator was also performed in toluene at room temperature. This polymerization led to very slow rate and low conversion as indicated from the SEC and ^1^H-NMR analysis (Figure S13). It was found that after almost three days (70 h), the conversion of *ε*-CL was only 25%, and the polydispersity increased to 1.15, while through ^1^H-NMR the degree of polymerization was found DP_PCL_ = 32. Similar results have been reported for the ROP of *ε*-CL using 1-pyrenebutanol or benzyl alcohol associated with *t*-BuP_1_, even at elevated temperatures [32].

## 4. Conclusions

In summary, an investigation of the anionic polymerization of styrene and 1,3-butadiene via high vacuum techniques was performed in the presence of phosphazene bases (*t*-BuP_1_, *t*-BuP_2_ and *t*-BuP_4_). Kinetic studies showed that the addition of the superbases prior the polymerization of styrene or butadiene increase the reaction rate due to the generation of highly reactive anionic species through the complexation with the lithium cation. The polymerization rate follows the order of [*t*-BuP_4_]/[*sec*-BuLi] >>> [*t*-BuP_2_]/[*sec*-BuLi] >> [*t*-BuP_1_]/[*sec*-BuLi] > *sec*-BuLi. The high basicity of *t*-BuP_4_ generates extremely reactive carbanions, which lead to high molecular weight and broad polydispersity due to the enhanced propagation rate or to the partial deactivation of the active centers by the *t*-BuP_4_. NMR studies currently in progress will enlighten the nature of the *t*-BuP_4_/*sec*-BuLi complex. On the other hand, when *t*-BuP_2_ was used, a decrease in the propagating site reactivity occurred, resulting in more controlled molecular characteristics. Using the weaker base (*t*-BuP_1_), the kinetic study showed that the polymerization rate was slightly increased compared with the conventional use of a metal-based initiating system (*sec*-BuLi) and that there is absolute control in the molecular weight and in the polydispersity. When polybutadiene was synthesized in the presence of PBs (*t*-BuP_2_, *t*-BuP_1_) in non-polar solvent, high vinyl content was obtained (45 wt % 1,2-microstructure) due to the delocalized negative charge. Finally, the one pot synthesis of polyester-based copolymers (PS-*b*-PCL and PS-*b*-PLLA) from the previously synthesized “living” homopolymers was feasible, by sequential copolymerization in the presence of *t*-BuP_2_ and *t*-BuP_1_, leading to a well-defined block of copolymers with desirable molecular characteristics. This study provides useful information towards the designing/synthesis of complex polyester-based polymeric materials.

## Figures and Tables

**Table 1:** Anionic polymerization of styrene and 1,3-butadiene in the presence of phosphazene bases.

Entry	Monomer	Phosphazene Base (PB)	PB/*sec*-BuLi ^a^	Time (h)	Conv. ^b^ (%)	M_n_ Targeted ^c^ (g/mol)	M_n_ SEC ^d^ (g/mol)	Ð ^d^
1	Styrene	*t*-BuP_4_	1:1	0.3	71.4	25,000	297,000	1.9
2	Styrene	*t*-BuP_2_	1:1	2	100	25,000	72,000	1.19
3	Styrene	*t*-BuP_2_	0.5:1	4	100	25,000	55,000	1.16
4	Styrene	*t*-BuP_1_	1:1	4	100	15,000	16,500	1.04
5	Styrene	*t*-BuP_1_	0.5:1	6	100	15,000	17,500	1.03
6	Styrene	--	--	6	100	15,000	15,800	1.02
7	Butadiene	*t*-BuP_2_	1:1	8	100	15,000	51,000	1.17
8	Butadiene	*t*-BuP_1_	1:1	12	100	8000	9300	1.03
9	Butadiene	--	--	16	100	10,000	11,300	1.02

^a^ Molar ratio of *sec*-BuLi and phosphazene base; ^b^ Conversion determined by ^1^H-NMR analysis; ^c^ Targeted number-average molecular weight calculated based on the feed ratio of monomer and initiator; ^d^ Number-average molecular weight calculated by SEC, using THF as solvent, calibrated with polystyrene standards.

**Table 2:** Sequential copolymerization of ε-caprolactone and L-lactide using the “living” PS.

Entry	Macro-Initiator ^a^	Monomer	Phosphazene Base (PB)	PB/Sec-BuLi ^b^	Time (h)	Conv. ^c^ (%)	M_n_ SEC ^d^ (g/mol)	Ð ^d^
1	(PS^−^Li^+^)_72k_	LLA	*t*-BuP_2_	1:1	16	--	72,000	1.20
2	(PS^−^Li^+^)_72k_	ε-CL	*t*-BuP_2_	1:1	16	--	72,000	1.20
3	(PS^−^Li^+^)_16.5k_	LLA	*t*-BuP_1_	1:1	16	--	16,500	1.04
4	(PS^−^Li^+^)_16.5k_	ε-CL	*t*-BuP_1_	1:1	16	--	16,500	1.04
5	(PSO^−^Li^+^)_72k_	LLA	*t*-BuP_2_	1:1	0.3	100	87,000	1.22
6	(PSO^−^Li^+^)_72k_	ε-CL	*t*-BuP_2_	1:1	17	70	82,000	1.23
7	(PSO^−^Li^+^)_16.5k_	LLA	*t*-BuP_1_	1:1	50	75	28,000	1.07
8	(PSO^−^Li^+^)_16.5k_	ε-CL	*t*-BuP_1_	1:1	70	25	20,000	1.15

^a^ The subscripts indicate the number-average molecular weight of the polystyrene (PS) macroinitiator in g mol^−1^, as given in Table 1; ^b^ Molar ratio of *sec*-Buli and phosphazene base used for the synthesis of the PS macroinitiator; ^c^ Conversion determined by ^1^H-NMR analysis; ^d^ Number-average molecular weight calculated by SEC, using THF as solvent, calibrated with polystyrene standards.

## Supplementary Materials

The following are available online at www.mdpi.com/2073-4360/9/10/538/s1, Figure S1: SEC traces obtained from withdrawn aliquots at 10 min and 20 min {[*t*-BuP_4_]/[*sec*-BuLi]:1/1, styrene}; Figure S2: ^1^H-NMR spectra after 30 min, 2 h and 4 h (100% conversion) {[*t*-BuP_2_]/[*sec*-BuLi]:0.5/1, styrene}; Figure S3: SEC traces obtained from withdrawn aliquots during the polymerization of styrene with [*t*-BuP_2_]/[*sec*-BuLi]:0.5/1; Figure S4: SEC traces obtained from withdrawn aliquots during the polymerization of styrene with [*t*-BuP_1_]/[*sec*-BuLi]:0.5/1; Figure S5: ^1^H-NMR spectra after 30 min, 2 h and 4 h (100% conversion) {[*t*-BuP_1_]/[*sec*-BuLi]:1/1, styrene}; Figure S6: ^1^H-NMR spectra after 2 h, 4 h and 6 h (100% conversion) {[*t*-BuP_1_]/[*sec*-BuLi]:0.5/1, styrene}; Figure S7. SEC traces obtained from withdrawn aliquots during the polymerization of styrene in the absence of phosphazene base; Figure S8: ^1^H-NMR spectra after 2 h, 4 h and 6 h (100% conversion) {styrene in the absence of phosphazene base}; Figure S9: ^1^H-NMR spectra after 2 h, 8 h and 12 h (100% conversion) {[*t*-BuP_1_]/[*sec*-BuLi]:1/1, 1,3-butadiene}; Figure S10: ^1^H-NMR spectra after 4 h, 8 h and 16 h (100% conversion) {1,3-butadiene in the absence of phosphazene base}; Figure S11: SEC traces obtained from withdrawn aliquots during the polymerization of 1,3-butadiene in the absence of phosphazene base; Figure S12: (A) SEC traces of PS (black line) synthesized with [*t*-BuP_2_]/[*sec*-BuLi]:1/1 and the corresponding PS-b-PLLA (red line). (B) ^1^H-NMR spectrum of the final diblock copolymer in CDCl_3_; Figure S13: (A) SEC traces of PS (black line) synthesized by [*t*-BuP_1_]/[*sec*-BuLi]:1/1 and of the corresponding PS-b-PCL (green line). (B) ^1^H-NMR spectrum of the final diblock copolymer in CDCl_3_.
