# Supplementary material for: Anionic Polymerization of Styrene and 1,3-Butadiene in the Presence of Phosphazene Superbases
Source: Polymers (Basel). 2017 Oct 21;9(10):538. doi: 10.3390/polym9100538 (PMC6418745; doi:10.3390/polym9100538)
Supplement: Supplementary file 1 [file polymers-09-00538-s001.pdf]

Anionic Polymerization of Styrene and 1,3-Butadiene in the Presence of Phosphazene Superbases

Konstantinos Ntetsikas[1], Yahya Alzahrany[1], George Polymeropoulos[1], Panayiotis Bilalis[1], Yves Gnanou[2], and Nikos Hadjichristidis[1,*]

1 *King Abdullah University of Science and Technology (KAUST), Physical Sciences and Engineering Division, KAUST Catalysis Center, Polymer Synthesis Laboratory, Thuwal, 23955, Saudi Arabia.*

2 *King Abdullah University of Science and Technology (KAUST), Physical Sciences and Engineering Division, Thuwal, 23955, Saudi Arabia.*

*Correspondence: nikolaos.hadjichristidis@kaust.edu.sa; Tel.: +966 (0) 544700134

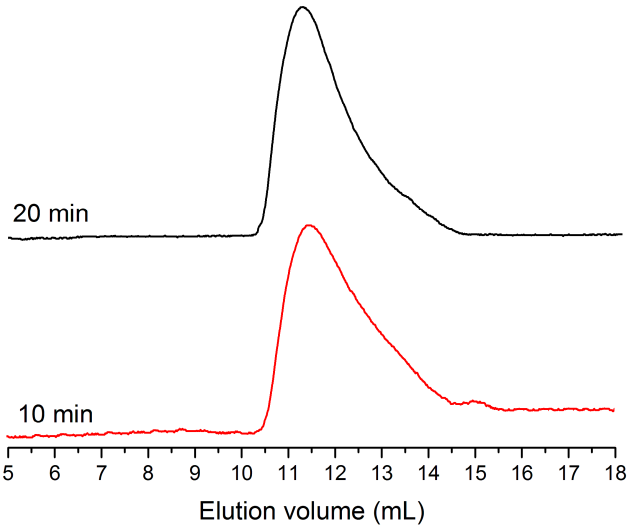

Figure S1. *SEC traces obtained from withdrawn aliquots at 10 min and 20 min {[t-BuP$_4$]/[sec-BuLi]:1/1, styrene}.*

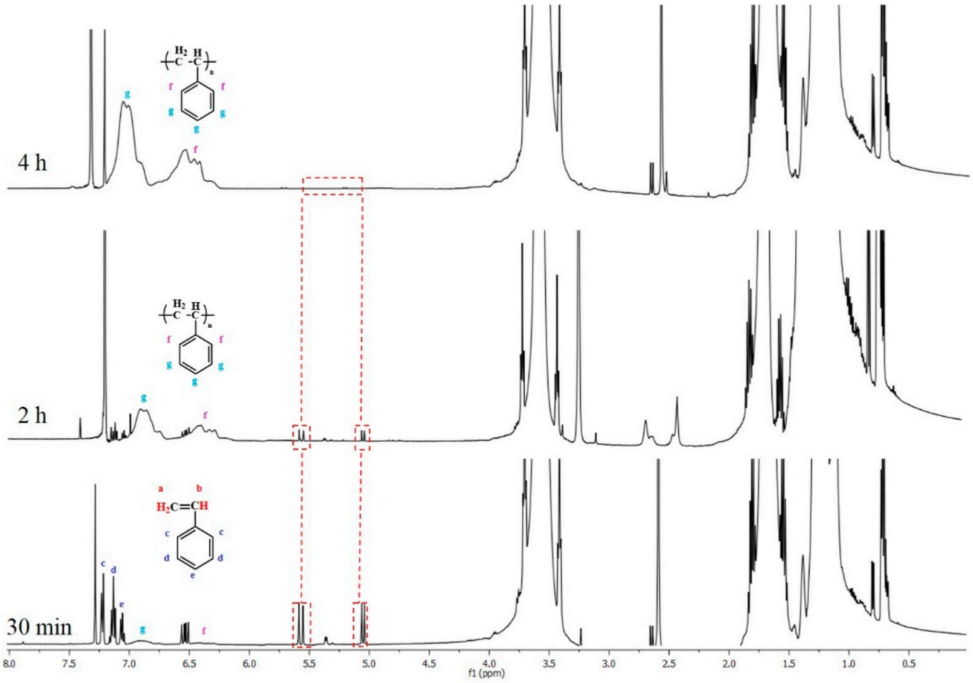

Figure S2. *^{1}H-NMR spectra after 30 min, 2h and 4h (100% conversion) {[t-BuP$_2$]/[sec-BuLi]:0.5/1, styrene}.*

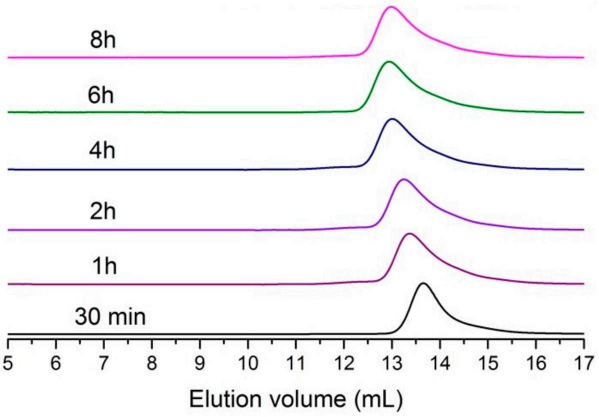

Figure S3. *SEC traces obtained from withdrawn aliquots during the polymerization of styrene with [t-BuP$_2$]/[sec-BuLi]:0.5/1.*

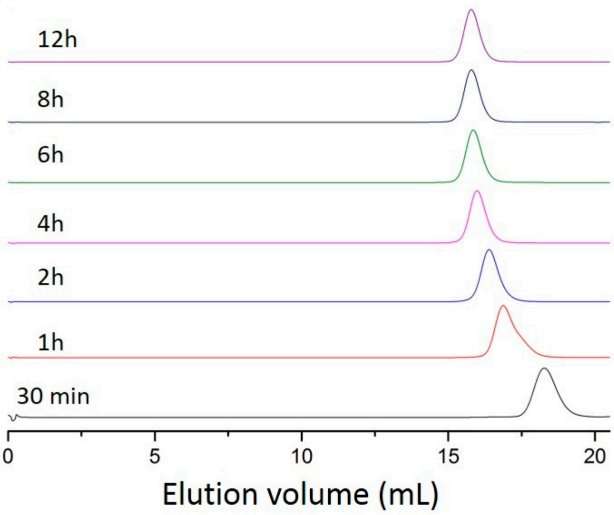

Figure S4. *SEC traces obtained from withdrawn aliquots during the polymerization of styrene with [t-BuP$_1$]/[sec-BuLi]:0.5/1.*

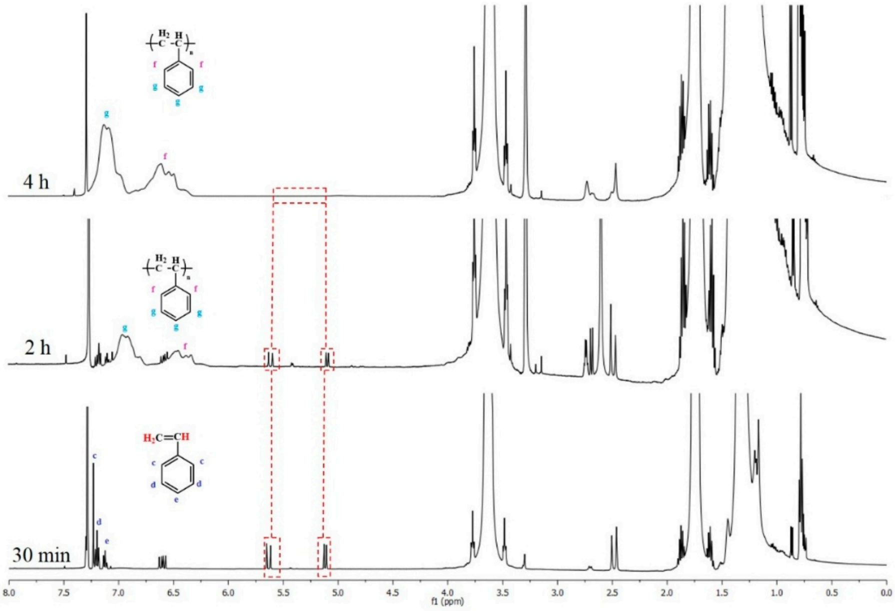

Figure S5. *^{1}H-NMR spectra after 30 min, 2h and 4h (100% conversion) {[t-BuP$_1$]/[sec-BuLi]:1/1, styrene}.*

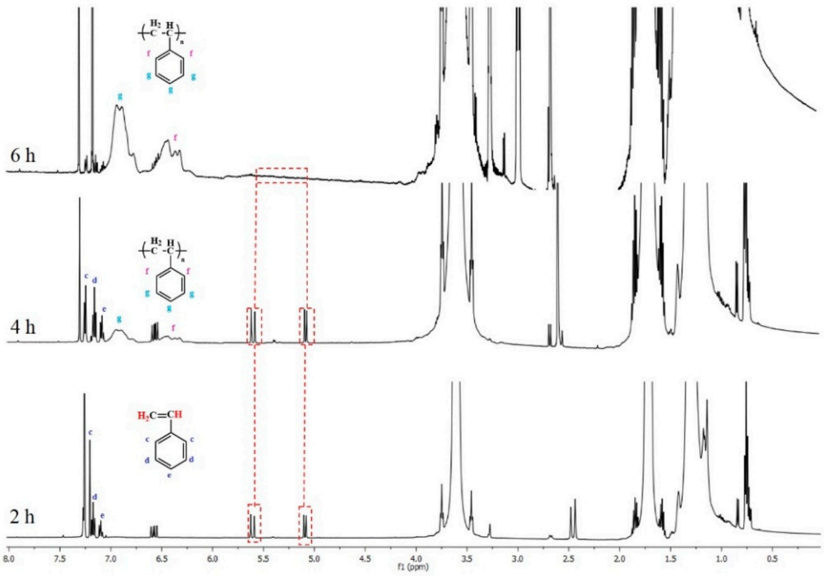

Figure S6. [1]*H-NMR spectra after 2h, 4h and 6h (100% conversion) {[t-BuP1]/[sec-BuLi]:0.5/1, styrene}.*

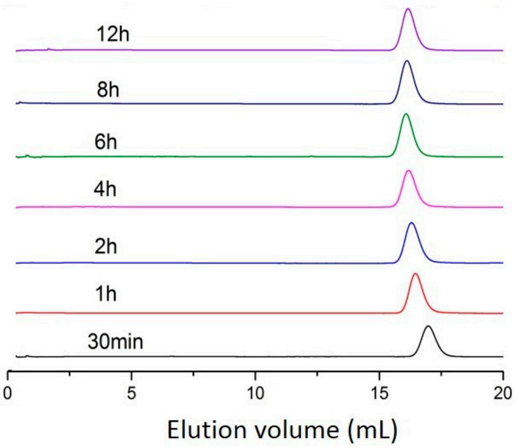

Figure S7. *SEC traces obtained from withdrawn aliquots during the polymerization of styrene in the absence of phosphazene base.*

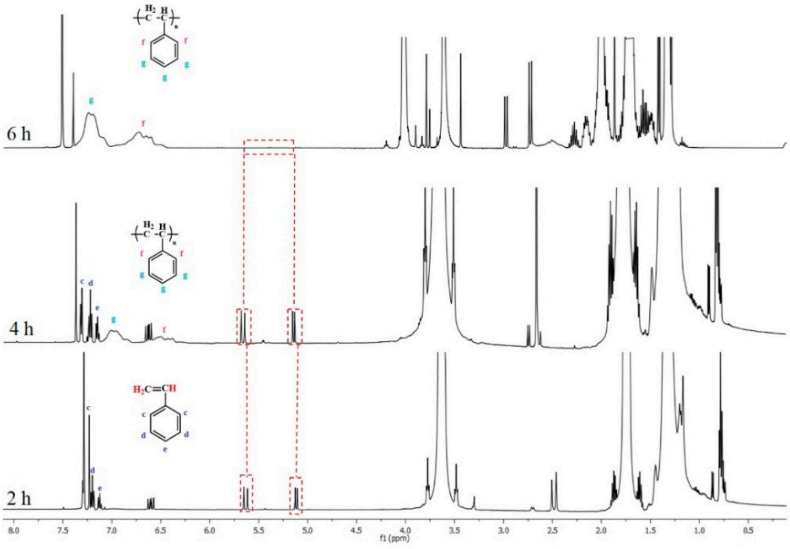

Figure S8. [1]*H-NMR spectra after 2h, 4h and 6h (100% conversion) {styrene in the absence of phosphazene base}.*

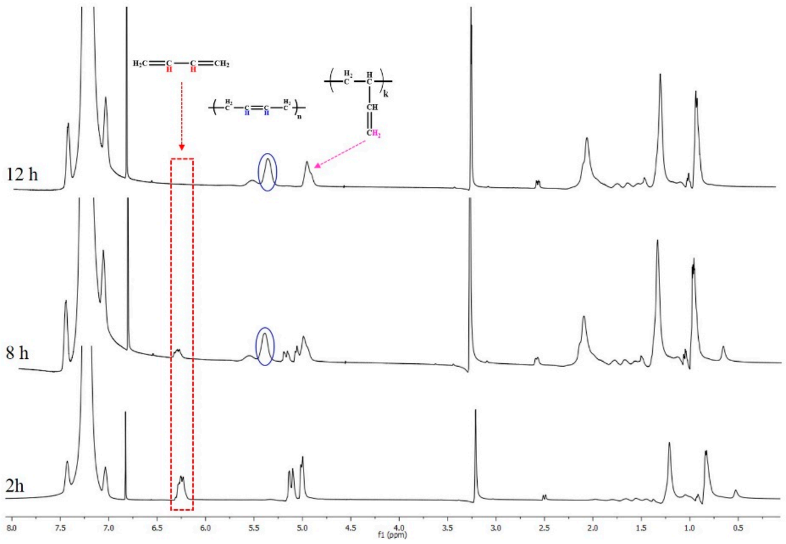

Figure S9. *^{1}H-NMR spectra after 2h, 8h and 12h (100% conversion) {[t-BuP$_1$]/[sec-BuLi]:1/1, 1,3-butadiene}.*

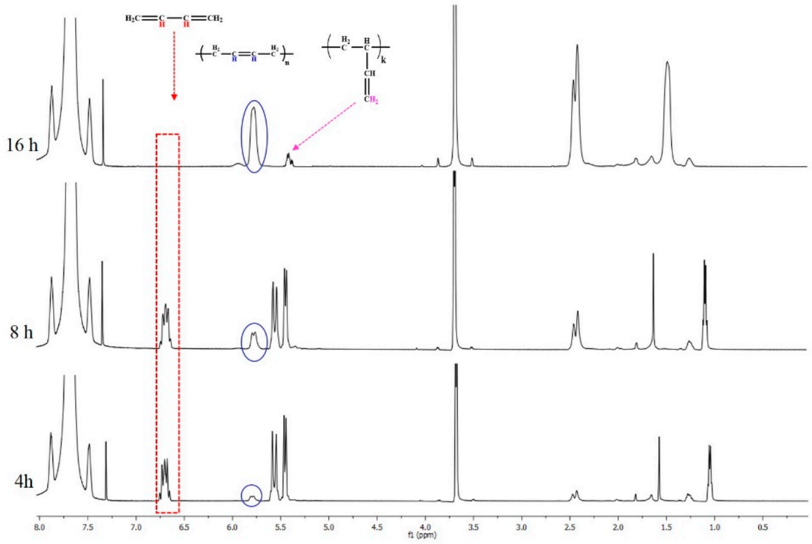

Figure S10. *^{1}H-NMR spectra after 4h, 8h and 16h (100% conversion) {1,3-butadiene in the absence of phosphazene base}.*

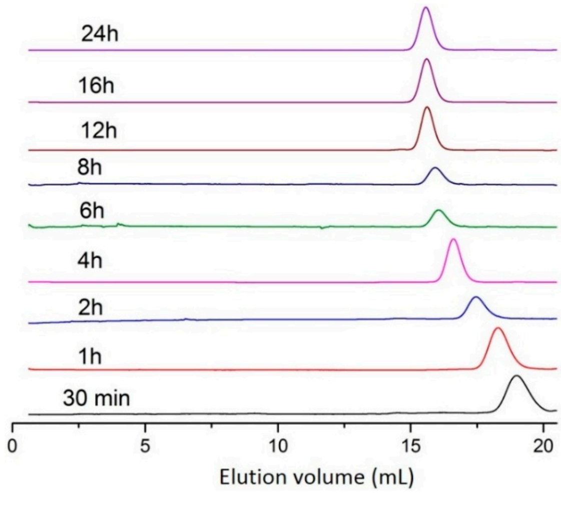

Figure S11. *SEC traces obtained from withdrawn aliquots during the polymerization of 1,3-butadiene in the absence of phosphazene base.*

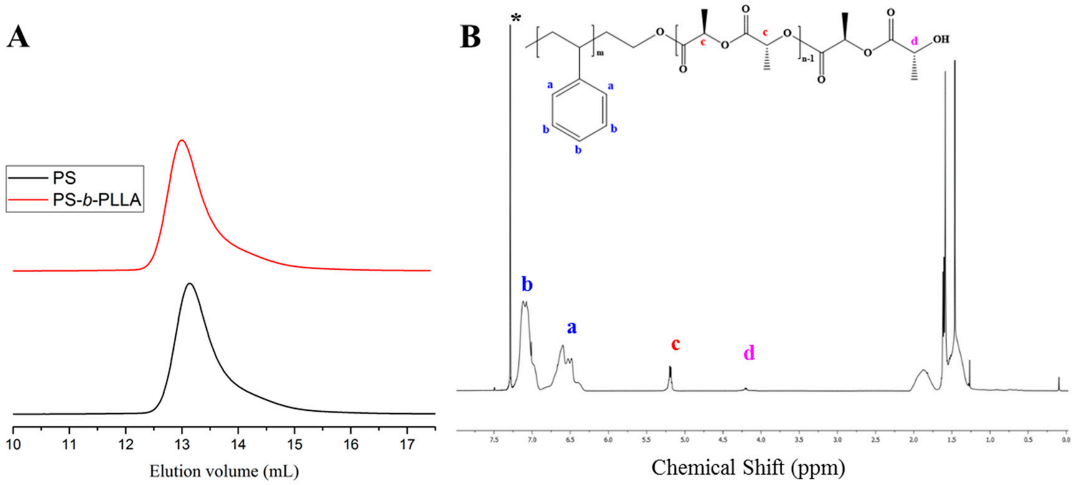

Figure S12. ***A***: *SEC traces of PS (black line) synthesized with [t-BuP$_2$]/[sec-BuLi]:1/1 and the corresponding PS-b-PLLA (red line).* ***B***: *^{1}H-NMR spectrum of the final diblock copolymer in CDCl$_3$.*

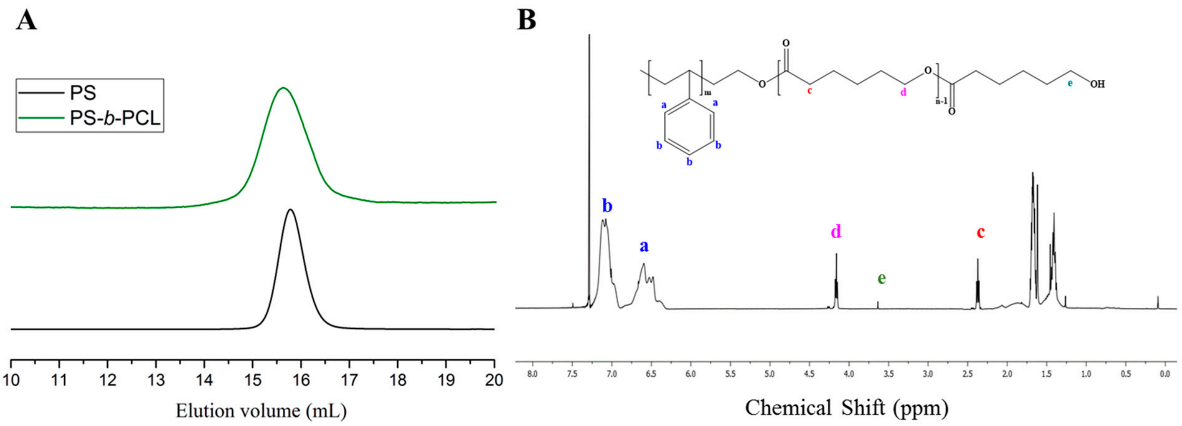

Figure S13. ***A***: *SEC traces of PS (black line) synthesized by [t-BuP$_1$]/[sec-BuLi]:1/1 and of the corresponding PS-b-PCL (green line).* ***B***: *^{1}H-NMR spectrum of the final diblock copolymer in CDCl$_3$.*
